# Evaluation of acoustic pulse and cold laser therapies for the treatment of dairy cows with subclinical mastitis under organic management conditions

**DOI:** 10.3389/fvets.2026.1856042

**Published:** 2026-07-24

**Authors:** Bhuwan Shrestha, Barbara W. Jones, Juan M. Piñeiro, Jennifer A. Spencer, Juan Velez, Narayan C. Paul, Guy Jodarski, Sushil Paudyal

**Affiliations:** 1 Department of Animal Science, Texas A&M University, College Station, TX, United States; 2 Department of Agriculture, Eastern Kentucky University, Richmond, KY, United States; 3 Texas A&M AgriLife Research and Extension Center, Stephenville, TX, United States; 4 Aurora Organic Farms, Boulder, CO, United States; 5 Texas A&M Veterinary Medical Diagnostic Laboratory, College Station, TX, United States; 6 CROPP Cooperative, Organic Valley, La Farge, WI, United States

**Keywords:** acoustic pulse, cold laser, mastitis, organic dairy, photobiomodulation

## Abstract

**Background:**

Mastitis remains a significant issue in dairy herds, yet antibiotic use is restricted under organic management conditions. There is a need to evaluate commercially available non-antibiotic mastitis treatment strategies.

**Objective:**

This study aimed to evaluate the effectiveness of two currently available mastitis treatment strategies: acoustic pulse therapy (APT) and cold laser therapy (CLT) on organic dairy farms.

**Methods:**

A total of 129 multiparous lactating crossbred cows with somatic cell counts (SCCs) >200,000 cells/mL were randomly assigned to one of three treatment groups: APT, CLT, and control (CON) at a commercial dairy farm under certified organic management. Cow-level milk samples were collected before milking and evaluated for SCC, fat%, protein%, lactose%, solids-not-fat%, and bacterial identification. Results from the dairy herd improvement association (DHIA) test were used to obtain individual milk production and cow removal information. The bacteriological cure rate, recovery rate, and cow survival were compared among treatment groups.

**Results:**

The CLT group had a higher bacteriological cure rate than the CON group, but only in cows with acute, contagious mastitis. Cows in the APT group tended to have lower bacterial counts than those in the CON group, regardless of inflammation status or chronic mastitis history. There was no statistically significant effect of treatment on cow recovery or survival.

**Conclusion:**

While there were some effects on bacteriological cure rate and bacterial counts in specific groups of cows, we were unable to demonstrate long-term benefits in terms of recovery rate, milk yield, or survival among cows affected by subclinical mastitis in organic settings.

## 1 Introduction

Mastitis is a common disease in dairy farms, affecting 42 and 14% of dairy cows globally with subclinical and clinical mastitis, respectively (1), and 13.8% of Holstein cows across 16 organic farms in the United States (2). Mastitis results in substantial economic losses for the dairy industry due to reduced milk yield, which accounts for 70% of the total losses (3), as well as discarded milk and increased culling of sick animals. Based on the presence of clinical signs, mastitis is generally classified into clinical and subclinical forms (4). Clinical mastitis is characterized by varying degrees and combinations of signs of inflammation, including redness and swelling of the udder and changes in milk appearance, such as clotting of milk (3). Subclinical mastitis is difficult to diagnose because of the apparent absence of visible signs (5), but it is more prevalent than clinical mastitis (6). Mastitis-causing bacteria can be either environmental or contagious. Environmental pathogens, such as *Streptococcus uberis*, *Streptococcus dysgalactiae*, CNS, *Escherichia coli,* etc., can be transmitted to cows during milking from contaminated environments. In contrast, contagious pathogens, such as *Staphylococcus aureus*, *Streptococcus agalactiae*, *Corynebacterium bovis,* etc., can be transmitted from cow to cow (7–10). Dairy farms usually implement antibiotic therapy to treat mastitis-infected cows (11), making mastitis solely responsible for approximately 80% of all antimicrobial usage in dairy farms (12). Consequently, mastitis is associated with public concerns related to antimicrobial resistance in dairy herds globally (13). Organic dairy farms have restrictions on the use of antibiotics as per the USDA National Organic Program (NOP) (14). In the US, there will be a permanent loss of the organic status of animals if the producer uses antimicrobials to treat the cows (15). Organic producers have limited options to treat mastitis-infected cows. Therefore, it is necessary to scientifically evaluate commercially available options for enhancing mammary gland health in cows, particularly those suitable for implementation in organic dairy herds.

Acoustic pulse therapy (APT) uses shockwave or low-power acoustic pulses that penetrate the tissue and distribute the pressure wave over a large area of the udder, producing a therapeutic effect. The APT influences the functions of cells and local homeostasis, thereby favoring the self-healing capabilities of tissue by promoting the proliferation, migration, and differentiation of stem cells (16). Shockwave therapy triggers early signals for angiogenesis by inhibiting endothelial cell apoptosis (17, 18), modulates inflammation through mechanisms such as the Toll-like receptor 3 (TLR3) pathway (19), and activates macrophages from a pro-inflammatory (M1) to an anti-inflammatory (M2) profile, enhancing tissue regeneration (16). Stem cells respond to the shockwave stimulus with increased expression of growth factors (TGF-*α* − 1) and production of nitric oxide (NO) and suppression of NF-k-B (nuclear factor kappa-light-chain-enhancer of activated B cell) and proinflammatory cytokines, such as IL-1β, IL-8, and IL-17 (20–23). In cases of mastitis, APT primarily focuses on restoring the health of the mammary gland by reducing the somatic cell count (SCC) to a normal level, which is below 250,000 cells/mL, by activating the self-healing capacities of tissues and modulating the inflammation (24).

The use of acoustic pressure pulse has been proven as a curative technique to treat musculoskeletal diseases in humans, dogs, and racehorses (25–27). Moreover, APT supports recovery from orthopedic inflammatory disorders (28, 29), erectile dysfunction (30, 31), and heart ischemia (32) in humans due to its angiogenesis and anti-inflammatory effects (29–32). There are very few studies that evaluate the effectiveness of the APT approach to managing udder health issues in dairy cows. Leitner et al. (33) reported that APT is as effective as antibiotics in treating clinical and subclinical mastitis in dairy cows in conventional farms. Nonetheless, to the best of our knowledge, APT has not been evaluated to treat cows in organic settings.

Cold laser therapy (CLT) is a common alternative therapy in veterinary medicine (34–36) and functions based on the low-intensity laser irradiation process called “photobiomodulation” (37, 38). Cold laser therapy, also known as low-level laser therapy, works through a photochemical mechanism in which light is absorbed by cellular chromophores, primarily cytochrome c oxidase (COX) in the mitochondria (39). This absorption increases electron transport, thereby boosting ATP production (40). Elevated ATP levels enhance the activity of ion transporters, including Na+/K + ATPase and Ca2 + pumps, which regulate critical cellular functions (41). The rise in ATP and the second messenger cyclic adenosine monophosphate (cAMP) triggers a cascade of biological effects, including increased cellular metabolism, tissue regeneration, reduced inflammation, and pain relief, without causing thermal damage to tissues, and it favors the healing of mastitis-affected udder (39). Low-level laser therapy predominantly uses red and near-infrared light (600–1,100 nm) for optimal tissue penetration (42).

Some studies have reported additional benefits of laser therapy when used as a supplemental treatment to antibiotics, with an increase in recovery rates of up to 24% in conventional farms (35). Although laser treatment is mainly used as an ancillary treatment (35, 38), few studies evaluate the effectiveness of laser therapy as the main treatment option to treat bovine mastitis (43–45). Therefore, we hypothesize that the two treatment options would have an effect on the subclinical udder infections compared with cows that received no treatment. The objective of this study was to evaluate the effect of APT and CLT on mastitis cure rate, somatic cell count, and mastitis-causing bacterial population (colony-forming units) in organic dairy cattle with subclinical mastitis infections. We also investigated whether treatment effects vary depending on the mastitis pathogen and the type of mastitis event, considering both their immediate and long-term durations.

## 2 Materials and methods

### 2.1 Research site and animals

The study was conducted at a commercial dairy farm under USDA-certified organic management in central Texas. The animals were housed in free stall barns, with concrete stalls bedded with sand, and milked in a double 18-parallel milking parlor twice a day. Cows had no access to pasture during the enrollment period. A total of 129 multiparous lactating crossbred cows with total cow-level somatic cell counts ≥200,000 cells/mL (46), confirmed by the Dairy Herd Improvement (DHI) test, were enrolled in the study between February and April 2023 and subsequently followed for three consecutive DHI tests. Overall, 10% of cows were primiparous, 21% were in parity 2, and 69% were in parity 3 and greater; 5% of cows were in early lactation (≤100 DIM), 34% were in mid-lactation (>100 to ≤200 DIM), and 61% were in late lactation (>200 DIM). The distribution and characteristics of cows in the treatment groups during enrollment are presented in Table 1.

**Table 1:** Descriptive representation of cow characteristics during enrollment (mean ± SD) of cows in treatment groups that received acoustic pulse (APT), cold laser (CLT), and control (CON) groups.

Item	Treatment			*p*-value
	APT (*n* = 47)	CLT (*n* = 40)	CON (*n* = 42)	
Lactation	3.3 ± 0.22	3.8 ± 0.24	3.4 ± 0.23	0.25
DIM	210 ± 12.8	220 ± 13.9	217 ± 13.6	0.19
LSSCC	5.9 ± 0.14	6.1 ± 0.15	5.8 ± 0.14	0.35
Milk production (kg/day)	19.6 ± 0.81	18.9 ± 0.87	19.2 ± 0.85	0.82

### 2.2 Treatments allocation

All treatment and animal handling protocols for this study were approved by the Texas A&M AgriLife Animal Care and Use Committee [IACUC #2022-026A]. Animals were assigned randomly to the three treatment groups: acoustic pulse therapy (APT, *n* = 47), cold laser treatment (CLT, *n* = 40), and control (CON, *n* = 42). The sample size was calculated using the OpenEpi tool, assuming a 40% bacteriological cure rate in the control group and a 70% bacteriological cure rate in the treatment group, with a 0.05% level of significance, indicating that 34 cows in each group would be able to detect a difference. A *post hoc* power analysis with G-Power 3.1^®^ using a repeated measures ANOVA yielded a power of 99% for the sample size used in this study. Cows in APT groups were treated with the acoustic pulse (wave) on two contralateral sides of the mammary gland uniformly to maximize the penetration of the waves to the interior udder tissues. Each side received 200 pulses (3.5 min/treatment) by using a commercial device, Armenta^®^(Armenta Inc., Israel), after a thorough ultrasound gel lubrication (24, 33, 47). This treatment protocol for APT used in this study differed from the company’s standard protocol, as we modified it to be used for the entire gland to have a comparable approach to CLT as opposed to the quarter-level application. The cows in the CLT group were treated with cold laser for 3 min on two contralateral sides of the mammary gland, ensuring maximal udder tissue exposure to the treatment using the Avant^®^ laser devices (Avant, CA, USA). The laser used was AVANT^®^ LZ30X, a class 3b laser that emits light at 600 nm and is approved for veterinary use as a low-level cold laser. The red light and infrared light settings were set to 150 and 30 timers, respectively, as recommended by the company, and used at around 30 cm from the quarter. Both treatments (APT and CLT) were applied on days 0, 3, and 5 of enrollment, after collecting the required milk samples, and follow-up was conducted on day 10 and day 25 of the treatment. Due to the nature of both treatments, the depth and breadth of treatment penetration are unknown, and application could not be restricted to an individual quarter. Consequently, treatments were administered to the entire mammary gland to evaluate effects at the gland level rather than at a specific quarter level. Cows in the control group (CON) did not receive either treatment and were managed using standard organic farm protocol, which includes complete milk harvest and monitoring for the development of clinical signs. It should be noted that cow allocation to the study groups was not based on bacterial status or on the number of infected quarters. Furthermore, all bacteriological analyses were conducted retrospectively. Therefore, potential baseline differences between groups at enrollment regarding pathogen distribution and infection burden cannot be excluded.

### 2.3 Microbiological analysis

The composite cow-level milk samples from all cows were collected on days 0, 3, 5, 10, and 25 of enrollment. The collected samples were immediately submitted to the DHIA laboratory (Texas DHIA laboratory, Stephenville, TX, USA) for evaluation of somatic cell counts (SCCs), fat%, protein%, lactose%, and solids-not-fat% (SNF). Additionally, composite aseptic milk samples for bacterial culture were collected from all cows after cleaning the teat ends with a cotton pad soaked in alcohol (70%) on day 0 (before treatment) and 25 (after treatment) of enrollment. Milk samples for bacterial culture were submitted to the Texas A&M Veterinary Medical Diagnostic Laboratory (TMVDL), Texas A&M University, for the culture, identification, and counts of the bacteria. For isolation and quantification of bacteria, milk samples were streaked onto 5% sheep blood agar, phenylethyl alcohol agar, and tergitol agar (Hardy Diagnostics, USA) plates and incubated aerobically with 10% CO_2_ at 37 °C for 48 h. All the culture plates were read at 24 h and 48 h for bacterial isolation. Initial identification of different bacteria was based on colony morphologies on plates and different biochemical test results, including oxidase, catalase, coagulase, indole, carbohydrate fermentation, and Gram staining. Identification of different bacteria at the genus or species level was based on matrix-assisted laser desorption-ionization time-of-flight mass spectrometry (MALDI-TOF MS; Bruker Daltonics, Germany) score results. A score of 2.3 to 3.0 is considered a highly probable species identification, and a score of 2.0 to 2.299 is considered a secure genus identification and probable species identification. The final identification of all bacterial isolates is based on the agreement between biochemical and MALDI-TOF MS results. After incubation, the colonies were counted (calculated as cfu/mL). A value of >10^5^ cfu/mL was assigned when the number of colonies was too numerous to count. Recorded milk production and SCC data for all cows were obtained from the farm management software program PCDART for the two DHI tests before treatment and the three DHI tests after treatment.

### 2.4 Outcome definition

Short-term (day 25 follow-up) and long-term (3 DHI tests follow-up) effects are considered to evaluate two levels of impact. A cow was considered bacteriologically cured if the bacterial species identified in the milk sample before treatment were not detected in the sample after treatment on day 25. Cows with bacterial culture results on enrollment (pre-treatment) and post-treatment were only included in the evaluation of bacteriological cure to evaluate CFU reduction. A cow was considered recovered when the SCC was lower than 200,000 cells/mL (LSSCC < 4.0) on two out of three DHI test days post-treatment (48).

### 2.5 Classification of mastitis events

Based on the level of inflammation, cows were classified as having acute and chronic mastitis. Cows with SCC levels above 400,000 cells/mL in 1 of the 3 DHI milk tests before the enrollment were considered to have acute subclinical mastitis, whereas cows with SCC of >400,000 cells/mL in more than 1 of the 3 DHI milk tests before the treatment were defined as chronic mastitis (24). Based on the bacteria involved, the study cows were also classified as having environmental mastitis and contagious mastitis events. The classification was based on the epidemiological origin and transmission pattern of the isolated pathogens. Environmental mastitis included cases caused by environmental pathogens, such as *Escherichia coli*, *Klebsiella* spp., and environmental *Streptococci*, whereas contagious mastitis included cases caused by pathogens primarily transmitted during milking, including *Staphylococcus aureus* and *Streptococcus agalactiae.*

### 2.6 Survival analysis

The survival of cows in the herd was analyzed as the long-term impact of the treatment and calculated based on the records of culling or death of animals within the follow-up period of 3 test days (average 132 days) after enrollment in the study.

### 2.7 Statistical analysis

The statistical analysis was performed in SAS (version 9.3; SAS Inst., Inc., Cary, NC, USA). The values for LSSCC were obtained to indicate linear scores of SCC by using the equation LSSCC = log_2_(SCC/100) + 3 (49). Bacterial colony-forming unit counts were log-transformed by using the equation logcfu = log_10_ (cfu + 1) to account for the zero cfu counts (11). For the bacterial culture analysis, samples with ≥3 isolates were considered contaminated and not included in the analysis (11, 50). The continuous outcome variables, including LSSCC, milk constituents (fat%, protein%, lactose%, and SNF), and bacterial colony-forming unit counts (logcfu), were analyzed using the mixed linear regression models (MIXED procedure). The model utilized included the fixed effects of treatment, day of treatment, and their interaction, parity, and DIM at enrollment. The baseline values (values of variables before the treatment) of all continuous variables were added to the model as covariate variables. Data were analyzed using cows within treatment as a random variable. For these repeated measurements, the specified term for repeated statements was day with cow (treatment) as the subject for the models. The compound symmetry covariance structure was used for the models based on the smallest Akaike Information Criterion. Tukey’s honest significant test for multiple comparisons was used in the model. The categorical variables, including bacteriological cure and recovery, and their odds were analyzed using binary logistic regression analyses (PROC GLIMMIX). The effect of the treatments on cow survival was evaluated by Cox’s proportional hazards using the PHREG procedure in SAS. The survival of the cows among the treatment groups was compared using Kaplan–Meier survival curves using the LIFETEST procedure in SAS. The significance of the effects was tested at *p* < 0.05 and the tendency at *p* < 0.10.

## 3 Results

### 3.1 Evaluating short-term impacts of treatment

#### 3.1.1 Cow-level LSSCC and milk constituents

The cow-level LSSCC analysis showed no effect of treatment on cow-level LSSCC between the treatment groups (Table 2). Similarly, there was no significant difference in cow-level fat%, lactose%, and SNF between the treatment groups. Among APT cows, the LSSCC was higher (*p* < 0.05) on day 3 than on day 10 of treatment. In the CON group, protein% was higher on day 25 than on day 3 (*p* < 0.05), day 5 (*p* < 0.001), and day 10 (*p* < 0.05) of treatment.

**Table 2:** Representation of cow-level LSSCC, fat%, protein%, lactose%, and solid not fat% (SNF) evaluations based on milk samples collected over time from subclinical mastitis-affected cows after three different treatments: acoustic pulse therapy (APT), cold laser therapy (CLT), and control (CON).

Variable	Day	APT	CLT	CON
LSSCC	3	4.9 ± 0.20^x^	4.0 ± 0.22	4.6 ± 0.22
	5	4.2 ± 0.20	4.0 ± 0.22	4.6 ± 0.21
	10	4.1 ± 0.20^y^	4.1 ± 0.22	4.3 ± 0.21
	25	4.2 ± 0.22	4.2 ± 0.23	4.3 ± 0.22
Fat	3	2.0 ± 0.18	1.8 ± 0.18	1.8 ± 0.20
	5	2.3 ± 0.18	2.2 ± 0.17	2.2 ± 0.19
	10	1.7 ± 0.18	2.1 ± 0.18	2.3 ± 0.19
	25	1.7 ± 0.18	1.9 ± 0.18	1.9 ± 0.20
Protein	3	3.3 ± 0.05	3.4 ± 0.05	3.3 ± 0.06^x^
	5	3.3 ± 0.05	3.4 ± 0.05	3.3 ± 0.06^x^
	10	3.3 ± 0.05	3.5 ± 0.05	3.3 ± 0.06^x^
	25	3.4 ± 0.06	3.5 ± 0.06	3.6 ± 0.06^y^
Lactose	3	4.2 ± 0.07	4.2 ± 0.07	4.3 ± 0.08
	5	4.3 ± 0.07	4.3 ± 0.07	4.1 ± 0.08
	10	4.2 ± 0.07	4.3 ± 0.07	4.2 ± 0.08
	25	4.2 ± 0.08	4.2 ± 0.07	4.2 ± 0.08
SNF	3	8.3 ± 0.11	8.5 ± 0.11	8.5 ± 0.12
	5	8.5 ± 0.11	8.5 ± 0.11	8.3 ± 0.12
	10	8.3 ± 0.11	8.6 ± 0.11	8.4 ± 0.12
	25	8.4 ± 0.12	8.5 ± 0.11	8.5 ± 0.12

^x,y^LSMean in a column within the same treatment for the same variable with different superscripts differ at *p* < 0.05.

#### 3.1.2 Bacterial dynamics

Gram-positive bacteria were identified in 34% of the culture samples, whereas 0.4% of the culture samples were Gram-negative, and 16% of the samples had no growth of bacteria (Table 3). *Staphylococcus chromogenes* (13%), *Staphylococcus aureus* (12%), and *Streptococcus uberis* (2%) were the top three frequently isolated pathogens from the milk cultures. The change in bacterial dynamics indicates an overall reduction in bacterial growth on day 25, with a sharp decrease in the number of positive cases of the top three bacteria in APT groups, specifically *Staphylococcus aureus* (7 vs. 4), CNS (13 vs. 7), and *Staphylococcus chromogenes* (11 vs. 6) (Table 3). While bacterial isolation remained unchanged in the untreated control group (0% reduction), both treatment groups achieved approximately a 50% reduction by day 25, highlighting a substantial treatment effect. This pattern was particularly evident for Gram-positive bacteria and *Staphylococcus aureus*.

**Table 3:** Distribution of bacterial species obtained from the culture of milk samples from cows with subclinical mastitis on days 0 and 25 of treatment, to evaluate the efficacy of acoustic pulse therapy (APT), cold laser therapy (CLT), and control (CON) for the treatment of subclinical mastitis.

Bacteria	Overall	Day 0 (*n*)				Day 25 (*n*)			
		APT	CLT	CON	Total	APT	CLT	CON	Total
Gram-positive	83 (34%)	24	10	16	50	13	5	15	33
*Staphylococcus aureus*	28 (12%)	7	2	7	16	4	1	7	12
*Streptococcus uberis*	6 (2%)	2	0	2	4	1	0	1	2
*Streptococcus dysgalacticae*	3 (1%)	0	1	1	2	0	0	1	1
*Corynebacterium* spp.	3 (1%)	0	2	0	2	0	0	1	1
*Bacillus* spp.	3 (1%)	0	1	1	2	0	0	1	1
*Staphylococcus hyicus*	1 (0.4%)	0	0	0	0	1	0	0	1
*Lactococcus* spp.	2 (0.8%)	2	0	0	2	0	0	0	0
*Streptococcus* spp.	1 (0.4%)	0	0	0	0	0	0	1	1
CNS	36 (15%)	13	4	5	22	7	4	3	14
*Staphylococcus chromogenes*	31 (13%)	11	4	4	19	6	3	3	12
*Staphylococcus* spp.	5 (2%)	2	0	1	3	1	1	0	2
Gram-negative	1 (0.4%)	0	0	0	0	1	0	0	1
*Pseudomonas* spp.	1 (0.4%)	0	0	0	0	1	0	0	1
No growth	23 (16%)	4	3	3	10	4	7	2	13
Mixed	102 (42%)	16	18	18	52	17	17	16	50
Contaminated	33 (14%)	3	8	5	16	4	7	6	17
Overall	242	47	39	42	128	39	36	39	114

#### 3.1.3 Bacteriological cure

The overall bacteriological cure rate (%) of APT was 28%, CLT was 37%, and CON was 21% (Table 4). There was no effect of treatments on bacteriological cure at day 25 of treatment. Based on the source of bacterial infection, CLT had a higher rate of cure for contagious bacteria than the CON group (*p* < 0.05). Based on the level of inflammation of mastitis, the bacterial cure for cows with acute subclinical mastitis was higher in the CLT group than in the CON group (*p* < 0.001).

**Table 4:** Results from mixed logistic regression analysis for overall, source of infection of bacteria, and at the inflammation level, evaluating odds of bacteriological cure at day 25, in cows treated with acoustic pulse (APT), cold laser (CLT), and control groups (CON).

Effect	LSM	SEM	OR (95% CI)	*p*-value
Overall				
APT	0.28	0.05	1.6 (0.42–6.26)	0.47
CLT	0.37	0.05	2.7 (0.71–10.80)	0.13
CON	0.21	0.05	Referent	
Environmental				
APT	0.30	0.07	1.0 (0.22–4.79)	0.96
CLT	0.36	0.07	0.9 (019–4.86)	0.96
CON	0.35	0.08	Referent	
Contagious				
APT	0.18^a,b^	0.08	1.5 (0.21–11.34)	0.65
CLT	0.38^a^	0.08	4.0 (0.61–26.66)	0.14
CON	0.15^b^	0.07	Referent	
Acute inflammation				
APT	0.43^a,b^	0.11	11.6 (0.32–411.7)	0.16
CLT	0.50^a^	0.10	17.5 (0.63–483.7)	0.08
CON	0.10^b^	0.10	Referent	
Chronic inflammation				
APT	0.25	0.05	1.00 (0.21–4.63)	1.00
CLT	0.32	0.06	1.56 (0.31–7.69)	0.58
CON	0.25	0.05	Referent	

^a,b^LSMean in a column within the same variable with different superscripts differ at *p* < 0.05.

#### 3.1.4 Bacterial count (logcfu)

**Figure 1:**
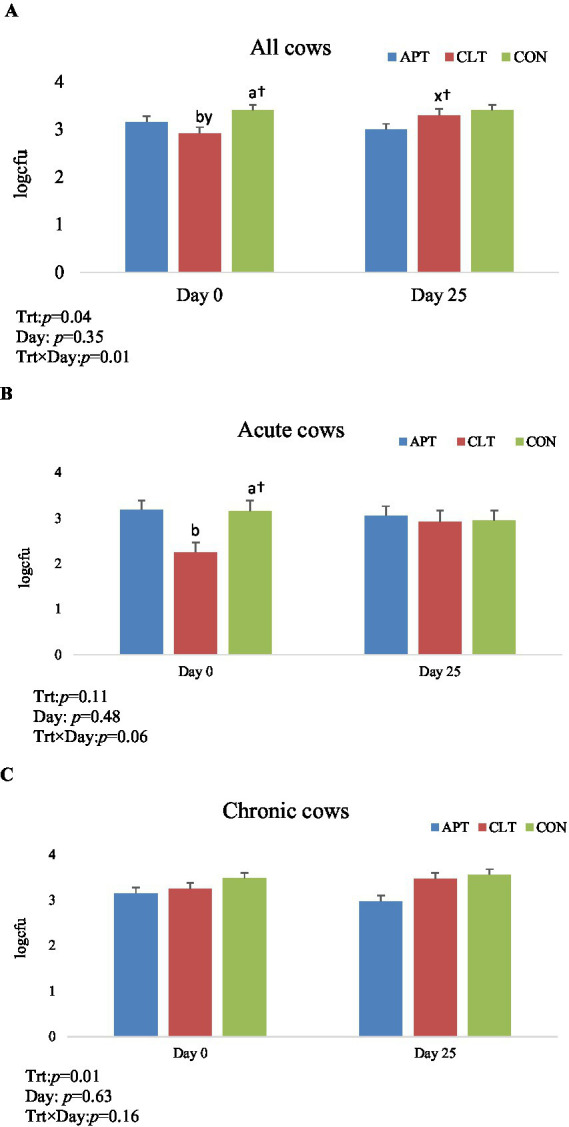
Representation of log-transformed bacterial colony-forming unit counts (logcfu) from cow-level milk samples in overall cows **(A)**, cows with acute mastitis **(B)**, and cows with chronic mastitis **(C)** in response to different treatments (APT, acoustic pulse therapy; CLT, cold laser therapy; and CON, control). Different superscript letters (a and b) indicate tendencies with significant differences between treatments on the same day (^†^*p* < 0.1). Different superscript letters (x and y) indicate tendencies for significant differences between days within the same treatment (^†^*p* < 0.1).

The analysis of the log-transformed bacterial colony-forming unit counts (logcfu) was based on the culture results of milk samples collected at days 0 and 25 of the treatment. The interaction effect of treatment × day of treatment on logcfu was observed when the data were analyzed with overall (regardless of inflammation) cow groups (Figure 1A). The CLT groups tended to have lower (*p* = 0.07) logcfu than the CON on day 0. Among the CLT group, the logcfu tended to be higher (*p* = 0.07) on day 25 than on day 0. Among cows with acute mastitis, the CLT group tended to have lower (*p* = 0.06) logcfu than the CON on day 0 (Figure 1B). Overall, the APT group tended to have lower (*p* = 0.06) bacterial counts than the CON group, whereas the CLT treatment did not demonstrate a statistically significant effect when compared with the CON and APT groups. Among cows with chronic inflammation, the APT group had lower (*p* < 0.05) bacterial counts than the CON group after treatment (Figure 1C).

### 3.2 Evaluating the long-term impact of treatment

#### 3.2.1 Recovery

The overall recovery percentage for cows was 13% for APT, 15% for CLT, and 14% for CON. No significant effect of treatments was observed in the overall recovery rate as well as the odds of recovery from the mastitis event (Table 5). We were also not able to detect a difference in the recovery based on the level of inflammation of mastitis (acute and chronic subclinical mastitis).

**Table 5:** Results from mixed logistic regression analysis with models containing overall cows and separated by severity level, evaluating odds of recovery (considered recovered when the SCC was lower than 200,000 cells/mL on two out of three DHI test days post-treatment) from inflammation when treated with acoustic pulses (APT), cold Laser (CLT), and control (CON) groups.

Effect	LSM	SEM	OR (95% CI)	*p*-value
Overall				
APT	0.13	0.05	0.97 (0.22–4.19)	0.97
CLT	0.15	0.06	1.13 (0.24–5.32)	0.86
CON	0.13	0.06	Referent	
Acute inflammation				
APT	0.11	0.13	0.7 (0.02–22.09)	0.86
CLT	0.33	0.16	3.0 (0.12–72.84)	0.47
CON	0.14	0.15	Referent	
Chronic inflammation				
APT	0.14	0.06	1.05 (0.19–5.65)	0.94
CLT	0.10	0.07	0.70 (0.09–5.06)	0.72
CON	0.13	0.07	Referent	

#### 3.2.2 Composite SCC (from pre-treatment and post-treatment DHI test)

The linear score of the somatic cell counts (LSSCCs) was evaluated based on the results of the DHI test (1 test day pre-treatment and up to 3 test days post-treatment). There was no difference in the LSSCC between the groups, both when analyzed overall (regardless of inflammation) and stratified by inflammation (acute and chronic) (Table 6).

**Table 6:** Representation of composite linear score of somatic cell counts (LSSCCs), daily milk yield (kg), milk fat%, milk protein% in overall cows, cows with acute mastitis, and cows with chronic mastitis response to different treatments (APT, acoustic pulse therapy; CLT, cold laser therapy; and CON, control) based on 1 pre- and 3 post-DHI test results from cows with subclinical mastitis.

Treatment	Test day	LSSCC	Milk yield	Fat	Protein
Overall					
APT	−1	5.9 ± 0.23	19.7 ± 0.87	4.2 ± 0.13^a^	3.2 ± 0.19
	1	5.6 ± 0.24	17.8 ± 0.87	4.1 ± 0.13	3.8 ± 0.19
	2	5.8 ± 0.24	14.5 ± 0.88	4.2 ± 0.14	3.3 ± 0.19
	3	5.9 ± 0.27	12.7 ± 0.94	4.1 ± 0.15	3.4 ± 0.22
CLT	−1	5.9 ± 0.25	18.9 ± 0.94	3.5 ± 0.15^by^	3.2 ± 0.20
	1	5.7 ± 0.27	16.6 ± 0.95	4.0 ± 0.15	3.4 ± 0.21
	2	5.3 ± 0.29	13.7 ± 0.99	4.4 ± 0.16	3.3 ± 0.23
	3	5.9 ± 0.30	10.3 ± 1.05	4.4 ± 0.17 ^x^	3.5 ± 0.25
CON	−1	5.9 ± 0.24	19.3 ± 0.92	4.1 ± 0.14	3.6 ± 0.20
	1	5.7 ± 0.25	16.5 ± 0.92	4.4 ± 0.14	3.5 ± 0.20
	2	5.6 ± 0.27	13.6 ± 0.95	4.1 ± 0.15	3.4 ± 0.21
	3	5.9 ± 0.32	10.3 ± 1.04	4.1 ± 0.17	3.4 ± 0.25
Acute inflammation					
APT	−1	5.5 ± 0.43	19.5 ± 1.72	4.3 ± 0.24	3.3 ± 0.10
	1	4.9 ± 0.44	18.3 ± 1.72	4.1 ± 0.24	3.3 ± 0.10
	2	3.6 ± 0.52	16.4 ± 1.82	4.3 ± 0.26	3.3 ± 0.11
	3	4.5 ± 0.63	15.4 ± 1.96	3.8 ± 0.28	3.4 ± 0.11
CLT	−1	5.6 ± 0.44	21.5 ± 1.98	3.3 ± 0.28	3.0 ± 0.12
	1	4.8 ± 0.48	20.6 ± 1.98	3.7 ± 0.28	3.2 ± 0.12
	2	3.5 ± 0.52	15.8 ± 2.06	4.1 ± 0.29	3.1 ± 0.12
	3	4.4 ± 0.63	13.3 ± 2.15	3.9 ± 0.31	3.3 ± 0.13
CON	−1	5.5 ± 0.50	22.3 ± 2.25	3.8 ± 0.32	3.2 ± 0.14
	1	5.1 ± 0.56	16.9 ± 2.37	4.4 ± 0.34	3.2 ± 0.14
	2	4.7 ± 0.56	14.4 ± 2.25	4.1 ± 0.32	3.3 ± 0.14
	3	6.3 ± 0.69	9.1 ± 2.71	3.9 ± 0.39	3.3 ± 0.15
Chronic inflammation					
APT	−1	6.0 ± 0.25	19.7 ± 1.00	4.2 ± 0.16	3.2 ± 0.24
	1	5.8 ± 0.26	17.7 ± 1.00	4.0 ± 0.16	4.1 ± 0.24
	2	6.1 ± 0.26	13.9 ± 1.00	4.2 ± 0.16	3.4 ± 0.24
	3	6.4 ± 0.30	11.9 ± 1.07	4.2 ± 0.18	3.4 ± 0.28
CLT	−1	6.1 ± 0.28	18.2 ± 1.06	3.6 ± 0.17^y^	3.2 ± 0.26
	1	6.1 ± 0.32	15.5 ± 1.08	4.1 ± 0.18	3.4 ± 0.27
	2	6.3 ± 0.33	13.1 ± 1.13	4.5 ± 0.19 ^x^	3.4 ± 0.29
	3	6.3 ± 0.33	9.9 ± 1.20	4.6 ± 0.20 ^x^	3.6 ± 0.33
CON	−1	6.1 ± 0.26	18.7 ± 1.00	4.1 ± 0.16	3.4 ± 0.24
	1	5.9 ± 0.27	16.4 ± 1.00	4.3 ± 0.16	3.5 ± 0.24
	2	5.9 ± 0.29	13.5 ± 1.04	4.1 ± 0.17	3.4 ± 0.26
	3	5.8 ± 0.34	10.5 ± 1.12	4.1 ± 0.19	3.4 ± 0.30

Different superscript letters (a and b) indicate significant differences between treatments on the same day (*p* < 0.05). Different superscript letters (x and y) indicate significant differences between days within the same treatment (*p* < 0.05).

#### 3.2.3 Milk production and constituents

No statistically significant effect of treatment on daily milk production or protein% (Table 6) was observed, either in the overall analysis (regardless of inflammation status) or when the data were stratified by inflammation status (acute and chronic), based on the results of the DHI test. However, the fat% in the milk was higher (*p* < 0.05) in the APT group than the CLT group on the test day before enrollment (Table 6). Among the CLT cows (regardless of inflammation), fat% was higher (*p* < 0.05) on the third test day than on the test day before enrollment. Similarly, among CLT cows with chronic mastitis, fat% was higher on the second (*p* < 0 0.001) and third (*p* < 0.001) test days after enrollment than on the test day before enrollment. However, treatment had no significant effect on fat% in cows with acute mastitis (Table 6).

#### 3.2.4 Survival in the herd

The overall rate of removal from the herd (culling or death) was 8.5% for APT, 10% for CLT, and 9.5% for CON. In total, 12 cows (four from each treatment group) were culled from the herd within the follow-up period of 3 test days after enrollment. The reason for culling was sold due to disease (*n* = 4), sold due to injury (*n* = 2), sold due to reproduction problems (*n* = 1), sold due to mastitis (*n* = 3), and culled due to death (*n* = 2). Each treatment group had one cow culled due to mastitis. The differences in the survival of the cows among treatments were analyzed using Kaplan–Meier survival curves and the Cox proportional hazards model. Similar survival curves were observed for all the treatment groups (log-rank test = 0.96). Similarly, there was no significant effect of treatment on the survival of cows based on the results from Cox’s proportional hazard model (*p* = 0.96).

## 4 Discussion

The study evaluated the short-term (up to 25 days after treatment) and long-term (up to 3 DHI test days after enrollment) impacts of two non-antibiotic treatment options: acoustic pulse therapy (APT) and cold laser therapy (CLT) for the management of subclinical mastitis.

We were unable to detect the effects of APT and CLT treatments on cow-level and quarter-level LSSCC in this study. Similarly, Stoffel et al. (45) did not observe a difference in LSSCC between laser-treated and untreated quarters when using low-energy laser irradiation as the primary treatment tool. However, others (24, 33, 47) reported decreases in LSSCC following APT compared with the CON group. We attribute the difference in these observations to the pathogens involved, management, and environment, as the organic farming system in the study may have cows that are immunologically different compared to conventional cows in other studies, contributing to the difference in response.

Another factor to consider when understanding these localized treatments is the difference in the location of the disease lesion and the tissue penetration of the treatments applied. Although Leitner et al. (47) claimed that APT covers approximately 3,487 cm^3^ of udder tissue as the treatment zone, the reach of treatment (depth) remains unknown. We tried to maximize penetration by applying treatment from two sides of the udder; however, the exact location of the inflammation within the mammary gland in these mastitis events is unknown. In addition, study factors, including the application of treatment at the cow level relative to the quarter level in other studies, could have resulted in variations in treatment responses reported across studies.

Neither APT nor CLT treatments demonstrate effects on cow-level fat%, protein%, lactose%, and SNF% in milk, indicating the absence of negative effects on milk parameters.

In our study, the overall APT group tended to have lower bacterial counts (logcfu) than the CON group. Additionally, cows affected with chronic subclinical mastitis that were treated with APT had lower bacterial counts than cows with chronic subclinical mastitis in the CON group. This suggests that acoustic pulse therapy could help with the management of chronic mastitis; however, these may be enhanced by optimizing the dosage and treatment frequency of APT. We observed a bacteriological cure rate of 28% for APT, which is lower than that observed by Blum et al. (24), who reported a bacteriological cure rate of 59% for APT. This difference may be due to differences in the pathogens involved, production system, treatment dose, and treatment frequency used in the respective studies. In our study, 34% of the isolates were Gram-positive bacteria, which may lead to these differences, as Blum et al. (24) found a higher overall cure rate for Gram-negative bacteria than for Gram-positive bacteria (*p* < 0.02).

The CLT group had a better bacteriological cure for cows infected with contagious mastitis-causing bacteria and for cows with acute subclinical mastitis compared with the CON group. Cows in the CLT group showed an impact on the cure of mastitis, especially acute mastitis events and contagious mastitis. This signifies that the CLT has the potential to be used for mastitis after treatment; however, not all cows and mastitis events benefit from the treatment.

We observed no statistically significant effects of treatment on the recovery of mastitis events under the definition and conditions of our study. We observed recovery rates of 13 and 14% in APT and CON, respectively, which were lower than those reported by Leitner et al. (47). These authors reported a recovery rate of 65.5% for APT and 35.6% for CON in conventional farms. No effects of treatment in the recovery of cows with subclinical mastitis indicate low treatment efficacy in the long term.

We did not detect a difference in milk yield in response to treatment, in contrast to Leitner et al. (47), who reported an increase in milk yield following APT compared with the CON group. The number of somatic cell counts (LSSCCs) did not differ among the treatment groups post-treatment. This may explain the absence of a difference in the milk yield among the treatments. In general, milk production is reduced due to elevated somatic cell counts, as increased somatic cell counts indicate more white blood cells in the milk to fight off pathogens causing infection and damaging the milk-producing cells (alveoli) (51, 52). Soumaya and Nagaty (44) reported an increase in the average milk yield of subclinical mastitis cows from 13 L to 18 L after treatment with low-light laser therapy twice daily for 5 days consecutively. The difference in the doses and frequency of the laser light used might have caused a difference in results regarding the milk yield. The milk yield of cows on all treatment groups (APT, CLT, and CON) decreased with the DHI test day, which was expected because of increased days in lactation.

The treatment groups demonstrated a similar survival (culling) percentage when following the cows up to three DHI tests after the treatment. Although there were several reasons for the exit of cows from the herds, we expected a lower culling due to mastitis in the APT and CLT treatment groups compared with the CON group. However, the result was different from our expectation, indicating that the treatments we used did not offer benefits in the long term.

The majority of the antimicrobials administered in dairy herds are used to treat mastitis and for dry cow therapy (12). The gold standard for the treatment of clinical mastitis is antibiotics, which yield a bacterial cure rate of 44 to 90% (11, 50, 53–55). However, the efficacy of the antibiotics depends on the type and dosage of the antibiotics used for the treatment. The use of antibiotics in subclinical mastitis varies depending on the farm protocol. Nonetheless, no antibiotic is allowed in the organic production system as per the national organic standards (56). Early detection and treatment of subclinical mastitis not only help to increase milk quality and milk yield but also help to prevent the progression of infection to clinical or chronic forms (57). The management of subclinical mastitis in organic herds helps to minimize the clinical cases that could lead to cows being removed from the farm due to culling or the use of antibiotics.

The spontaneous bacteriological cure refers to the cure due to the immune ability of the host, which varies widely for the mastitis-causing pathogens (58). The estimated spontaneous cure rate for *Staphylococcus aureus* is 0 to 5%, for non-aureus *Staphylococcus* is 55 to 60%, and for *Escherichia coli* is approximately 90% (50, 58). This indicates that in cases of mastitis by *E. coli*, cows could self-recover even without treatment, whereas in cases of *S. aureus*, some intervention is important to prevent the progression of subclinical cases to clinical cases. Therefore, the need and effectiveness of treatment are dependent on the type of mastitis-causing pathogen, farm management, and goals. Although the evaluation of treatment effect on individual mastitis pathogens would yield conclusions at the pathogen level, such an approach would result in significant logistical and financial constraints in this study to balance cows by pathogen groups. However, the data presented suggest that with more research and refinement, these treatment options could be adopted as management approaches and have potential as ancillary treatments in conventional herds to optimize or supplement existing treatments.

An additional methodological consideration is that the treatment was applied to the entire udder rather than specifically to the infected quarter and its teat. Because the bovine udder consists of four separate mammary glands, this approach effectively reduced the treatment exposure delivered to the infected quarter to approximately one-quarter of that achieved with targeted application. Consequently, the infected quarter may not have received the intended dose. Moreover, according to the company’s protocol, when two mammary glands are affected, each gland should receive a full treatment application. Thus, in cases involving two infected glands, the recommended protocol would result in approximately twice the treatment exposure compared with the approach used in the present study. Therefore, the observed efficacy was achieved under conditions that likely provided a lower-than-recommended treatment dose.

We used composite (cow-level) milk samples rather than specific quarter-level samples for bacteriological analysis and SCC evaluation because of the nature of both treatments, which were applied at the udder level. The effects of these treatments cannot be confined to a single quarter, with the unknown penetration depth and spread of the waves and light. However, because mastitis is a quarter-based infection, composite sampling can dilute the SCC of an infected quarter with milk from healthy quarters or mask the cure of one quarter if another becomes newly infected (48, 59). Consequently, the bacteriological cure reported here represents a cow-level status and may not capture the dynamic infection status of individual mammary quarters. However, our approach should provide an understanding of the overall impact on the udder to manage mastitis at the cow level. In addition, our protocol maintained the exact total therapeutic dosage validated by Blum et al. (24) and Leitner et al. (33, 47), who prescribed 400 total pulses delivered across three sessions. While the referenced studies used this dosage globally, we strategically adapted the delivery to the cow level to evaluate a generalized therapeutic approach targeted to the entire mammary gland. Because both methods serve as non-specific treatments aimed at reducing overall somatic cell counts, this adaptation directly aligns with our core objective of comparing CLT and APT treatment. Future research should utilize quarter-level sampling and treatment to precisely define therapeutic efficacy and spontaneous clearance.

## 5 Conclusion

While we identified effects on cure rate and bacterial counts in specific groups of cows, both treatments failed to demonstrate long-term benefits in recovery rate, milk yield, or survival among cows affected by subclinical mastitis in organic settings. Further research is needed to optimize the dose and duration of treatment to achieve more consistent and sustained beneficial outcomes on dairy farms.

## Data Availability

The original contributions presented in the study are included in the article/supplementary material, further inquiries can be directed to the corresponding author.
